# CHD8 regulates gut epithelial cell function and affects autism-related behaviors through the gut-brain axis

**DOI:** 10.1038/s41398-023-02611-2

**Published:** 2023-10-02

**Authors:** Ipsita Chatterjee, Dmitriy Getselter, Nasreen Ghanayem, Ram Harari, Liron Davis, Shai Bel, Evan Elliott

**Affiliations:** Azrieli Faculty of Medicine, Bar Ilan University, Safed, 13215 Israel

**Keywords:** Molecular neuroscience, Autism spectrum disorders

## Abstract

Autism is a neurodevelopmental disorder characterized by early-onset social behavioral deficits and repetitive behaviors. Chromodomain helicase DNA-binding protein (CHD8) is among the genes most strongly associated with autism. In addition to the core behavioral symptoms of autism, affected individuals frequently present with gastrointestinal symptoms that are also common among individuals harboring mutations in the gene encoding CHD8. However, little is known regarding the mechanisms whereby CHD8 affects gut function. In addition, it remains unknown whether gastrointestinal manifestations contribute to the behavioral phenotypes of autism. The current study found that mice haploinsufficient for the large isoform of *Chd8* (*Chd8L*) exhibited increased intestinal permeability, transcriptomic dysregulation in gut epithelial cells, reduced tuft cell and goblet cell counts in the gut, and an overall increase in microbial load. Gut epithelial cell-specific *Chd8* haploinsufficiency was associated with increased anxiety-related behaviors together with a decrease in tuft cell numbers. Antibiotic treatment of *Chd8L* haploinsufficient mice attenuated social behavioral deficits. Together, these results suggest Chd8 as a key determinant of autism-related gastrointestinal deficits, while also laying the ground for future studies on the link between GI deficits and autism-related behaviors.

## Introduction

Autism is a neurodevelopmental disorder characterized by early-onset social behavioral deficits and repetitive behaviors [1]. In addition to these core symptoms, gastrointestinal (GI) symptoms, such as constipation, bloating, abdominal pain and diarrhea [2], are common among individuals with autism [3], with a positive correlation found between the degree of GI disturbances and severity of autism [4, 5] Constipation, for example, affects an estimated 20-33.9% of these patients [6]. In addition, many studies have reported on changes in the composition of the gut microbiome in both patients [7–9] and in animal models of autism [10]. In one study, an estimated 43% of individuals with autism exhibited abnormal intestinal permeability [11], which aligned with a report published by De Magistris et al. that found that autistic individuals (36.7%) and their relatives (21.2%) were more likely to exhibit abnormal intestinal permeability as compared with typically developing subjects (4.8%) [12]. Differences in intestinal permeability can be a direct result of changes in mucus secretion and/or the thickness of the mucus layer, which serves as a critical barrier between luminal antigens and host tissues. Gut epithelial cells are primarily responsible for maintaining proper permeability and mucus production, the latter of which is mediated by the goblet cells [13].

Chromodomain helicase DNA-binding protein (*CHD8*) is one of the genes most strongly associated with autism [14]. Several studies have reported links between various severe *CHD8* mutations and the incidence of autism [15–18]. Encoded on chromosome 14q11.2, CHD8 is a chromatin-remodeling factor [19] that binds to β catenin [20] and regulates the Wnt signaling pathway [21]. Wnt signaling is an intracellular pathway involved in many processes and has specifically been implicated in the regulation of proliferation during neurodevelopment [22]. Nearly 80% of patients harboring *CHD8* mutations have also been found to suffer from gastrointestinal disturbances, with 60% reporting constipation. Bernier et al. found that downregulation of *CHD8* expression in zebrafish results in slower gastrointestinal motility and reduced post-mitotic enteric neuron counts [15].

Despite these intriguing reports, little is known about the direct molecular link between CHD8 and gut function. Furthermore, it remains to be determined if changes in gut function impact autism-associated behaviors. To address this knowledge gap, this study employed a mouse model harboring the heterozygous knockout of the large isoform of CHD8 (CHD8L). While a complete *Chd8L* knockout has been reported to be lethal, Katayama et al. [23]. found that *Chd8L*^+/−^ mice exhibited increased brain weight and brain volume relative to control mice, consistent with macrocephaly observed in autistic individuals with *CHD8* mutations [15]. *Chd8L*^+/−^ mice also presented with anxiety-like behaviors and social interaction deficits, as manifested by a reduction in the duration of active contact relative to controls. In parallel, they exhibited shorter intestines and slower GI motility [23]. The current study sought to compare functional, cellular and molecular characteristics of the gut of *Chd8L*^+/−^ vs. wild type (WT) mice, and to assess the possibility of a link between changes in GI signatures and the anxiety-like behavior that arises as a consequence of *Chd8* haploinsufficiency.

## Results

### Increased gut permeability in *Chd8L*^+/−^ mice

**Fig. 1:**
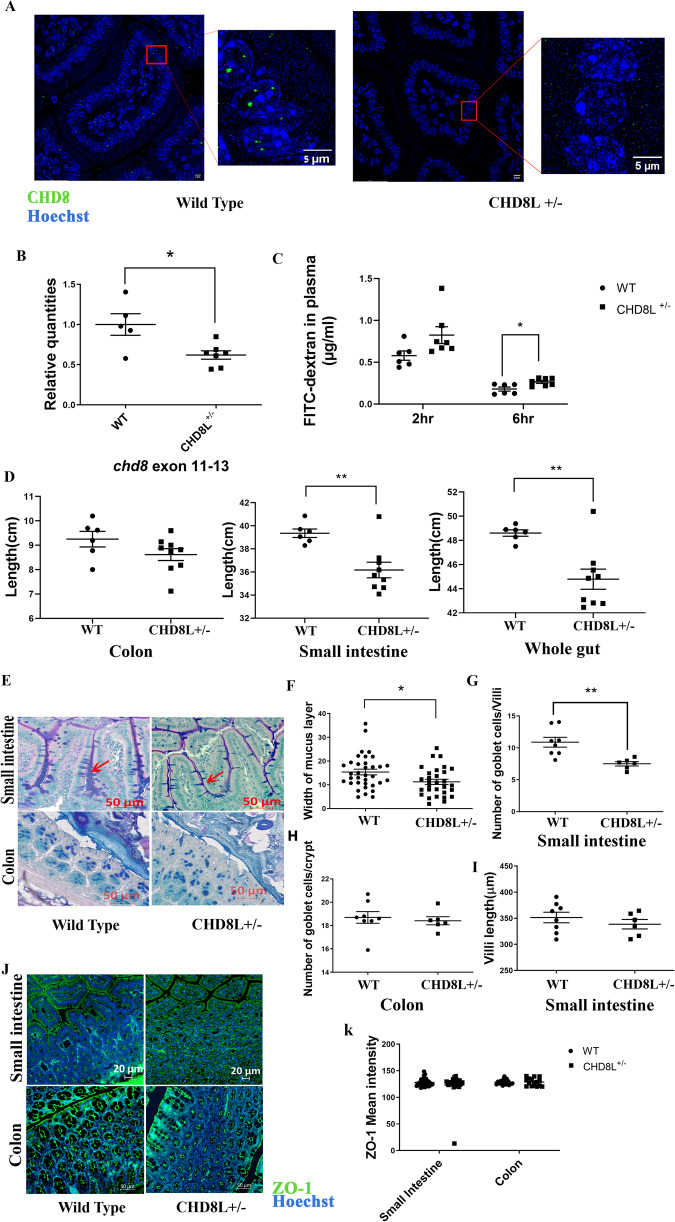
Functional and morphological analysis of gut epithelial layer in *Chd8L* +*/*− mice. **A** Representative images of CHD8 staining (green) in wild type (WT) and *Chd8L*^+/−^ gut epithelial layer. Hoechst dye (blue) was applied as a nuclear counterstain. The magnified area is marked with a red box. **B** Real-time PCR analyses of *Chd8* exons 11–13, revealing a reduction in relative Chd8 levels in *Chd8L*^+/−^ mice (*p < 0.05, two-tailed unpaired t-test; WT: *n* = 5, *Chd8L*^+/−^: *n* = 7). **C** Plasma FITC-dextran levels measured 2 h and 6 h post-gavage (*p < 0.05; WT: *n* = 10, *Chd8L*^+/−^: *n* = 15). **D** Colon, small intestine and whole-gut lengths of WT (*n* = 6) and *Chd8L*^*+/−*^ (*n* = 9) mice (**p < 0.01). **E** Representative periodic acid Schiff staining of gut epithelial layer from WT and *Chd8L*^+/−^ mice. Goblet cells are indicated by the red arrows. Red lines indicate the thicknessof the mucus layer. **F**–**I** Morphological analysis of the (**F**) mucus layer thickness (*p < 0.05, two-tailed unpaired t-test; WT: *n* = 35, *Chd8L*^+/−^: *n* = 30), (**G**) goblet cell count per villus (**p < 0.01, two-tailed unpaired t-test; WT: *n* = 8, *Chd8L*^+/−^: *n* = 6), (**H**) goblet cell count per crypt, and (**I**) villi length. **J**, **K** ZO-1 staining of colon and small intestine samples from WT (*n* = 5 each) and *Chd8L*^+/−^ mice (*n* = 5, colon; *n* = 4, small intestine). Representative ZO-1 staining results are shown (**J**) together with mean ZO-1 intensity (**K**). Data are means ± SEM.

Before exploring the relationship between CHD8 and GI function, the *Chd8L*^+/−^ experimental model was first validated. Immunohistochemical staining confirmed that CHD8 protein levels were reduced in the GI tract of *Chd8L*^+/−^ mice relative to WT controls (Fig. 1A). In *Chd8L*^+/−^ mice, exons 11–13 of the *Chd8* gene are deleted. Real-time PCR analyses measured a significant downregulation of *Chd8* exons 11–13 (Fig. 1B) in the gut epithelial cells of *Chd8L*^+/−^ mice as compared with WT controls. In parallel, gut epithelial cells of the haploinsufficient mice exhibited increased expression of *Chd8* exon 1 (Supplementary Fig. 1A), suggestive of an attempt to compensate for the loss of CHD8L.

To assess intestinal permeability, mice were gavaged with fluorescein isothiocyanate-conjugated dextran (FITC-dextran) and plasma levels of FITC-dextran were measured 2 h or 6 h thereafter. At 6 h post-gavage, plasma FITC-dextran levels were elevated in *Chd8L*^+/−^ mice relative to WT controls (Fig. 1C), suggesting increased intestinal permeability. While the length of both the small intestine and of the overall intestines was shorter in *Chd8L*^+/−^ mice as compared with their WT counterparts, no differences in colon length (Fig. 1D) or in stool transit time were measured between the genotypes (Supplementary Fig. 1B). Our results are consistent with the previous study by Katayama et al., reporting a decrease in intestinal length in Chd8L +/− mice [23].

### *Chd8L*^+/−^ mice exhibit altered gut morphology

AB-PAS staining of small intestine and colon tissue samples excised from 8-week-old *Chd8L*^+/−^ mice and wild type mice identified a reduction in colon mucus layer width (Fig. 1E, F) and in the number of mucus-producing goblet cells in the small intestine (Fig. 1G), but not in the colon (Fig. 1H) of *Chd8L*^+/−^ mice. No differences in villi length were detected between the two animal groups (Fig. 1I). Staining for the tight junction marker ZO-1 in 8-week-old mice revealed no differences in tight junction morphology or tight junction marker ZO-1 intensity between wild type and *Chd8L*^*+/−*^ mice (Fig. 1J, K). Signs of altered gut morphology, particularly in the mucus layer and goblet cell count, were not apparent in 4-week-old *Chd8L*^*+/−*^ mice (Supplementary Fig. 2).

### Transcriptomic analysis of *Chd8L*^+/−^ mouse gut epithelial cells

**Fig. 2:**
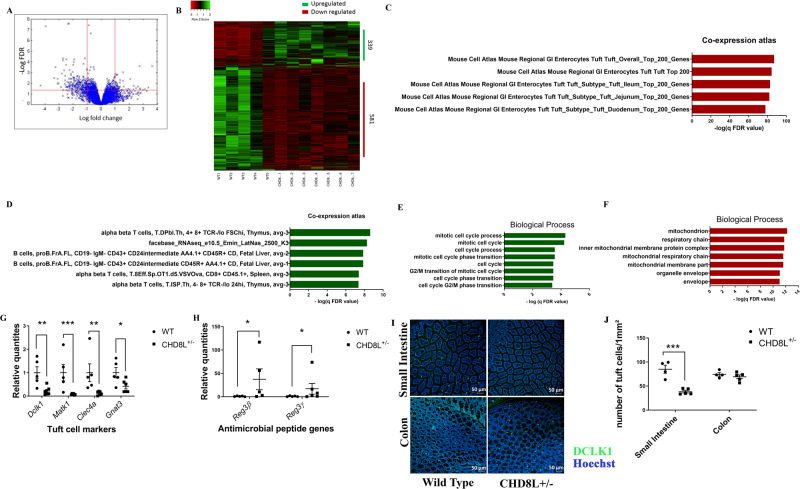
Transcriptomic analysis of gut epithelial cells in *Chd8L* + */*− mice. **A** A volcano plot highlighting differentially expressed gene distributions in *Chd8L*^+/−^ gut epithelial cells. Each point represents genes in *Chd8L*^+/−^ mice plotted against the level of statistical significance (−log10 adjusted *p*-value) and fold-change in expression (log2 (*Chd8L*^+/−^ vs. WT). **B** Heatmap showing the 920 genes differentially expressed in *Chd8L*^+/−^ mouse gut epithelial cells compared with WT littermates (FDR-adjusted *p* ≤ 0.05). **C**–**F** Gene Ontology analyses of differentially expressed genes, including co-expression analyses of downregulated (**C**) and upregulated (**D**) genes, as well as biological process term enrichment results for upregulated (**E**) and downregulated (**F**) genes. **G** Real-time PCR analyses of tuft cell markers, including *Dclk1* (**p < 0.01), *Matk1* (***p < 0.001), *Clec4a* (**p < 0.01) and *Gnat3* (*p < 0.05) (unpaired two-tailed t-test, WT: *n* = 5, *Chd8L*^+/−^: *n* = 7). **H** Real-time PCR analyses of the antimicrobial peptide genes *Reg3*γ (*p = 0.05) and *Reg3*β (*p < 0.05) (unpaired two-tailed t-test, WT: *n* = 5, *Chd8L*^+/−^: *n* = 7). **I** Representative images of immunohistochemically stained colon and small intestine tissue samples from WT (*n* = 4) and *Chd8L*^+/−^ (*n* = 5) mice. Samples were stained for DCLK1 (green) with Hoechst (blue) as a nuclear counterstain. **J** Significant decreases in the number of tuft cells per mm^2^ were evident in the small intestine of *Chd8L*^+/−^ mice relative to WT mice (***p < 0.001; unpaired two-tailed T-test). Data are means ± SEM.

To assess the impact of *Chd8L*^+/−^ on transcriptional activity in the gut, comparative whole transcriptomic sequencing was performed on gut epithelial cells extracted from WT and *Chd8L*^+/−^ mice. Strikingly, over 900 genes were differentially expressed, including 581 that were downregulated and 339 that were upregulated in *Chd8L*^+/−^ gut epithelial cells (Fig. 2A, B; Supplementary Tables 1, 2). Gene Ontology (GO) enrichment analyses indicated that markers of tuft cells, a subtype of gut epithelial cells that induce type 2 immune responses and promote goblet cell development [24], were overrepresented in the downregulated genes, while upregulated genes were enriched for genes associated with immune cells and the immune response (Fig. 2C, D, Supplementary Table 3). More specifically, downregulated and upregulated genes were enriched for GO biological process terms related to mitochondrial function and the cell cycle, respectively (Fig. 2E, F, Supplementary Table 3). Comparison of the list of downregulated genes to a recently published list of subtypes of tuft cells identified by single-cell sequencing, found that 22 of the downregulated genes were among the list of 25 markers for type 2 tuft cells [25]. RT-PCR analyses further confirmed the downregulation of four tuft cell marker genes in *Chd8L*^*+/−*^ samples (Fig. 2G). RNA-seq analyses, verified by RT-PCR, identified the key antimicrobial peptides Reg3β and Reg3γ as two of the most highly upregulated genes in *Chd8L*^+/−^ mice (Fig. 2H). Immunohistochemical staining for the tuft cell marker protein DCLK1 (Fig. 2I), measured a decrease in DCLK1-positive cells in the small intestine of *Chd8L*^+/−^ vs. WT mice, without any corresponding change in the colon (Fig. 2J). These findings suggest that the reduced expression of tuft cell marker genes stemmed from the overall drop in tuft cell counts in the haploinsufficient mice.

### *Chd8L*^+/−^ mice exhibit an altered gastrointestinal microflora

**Fig. 3:**
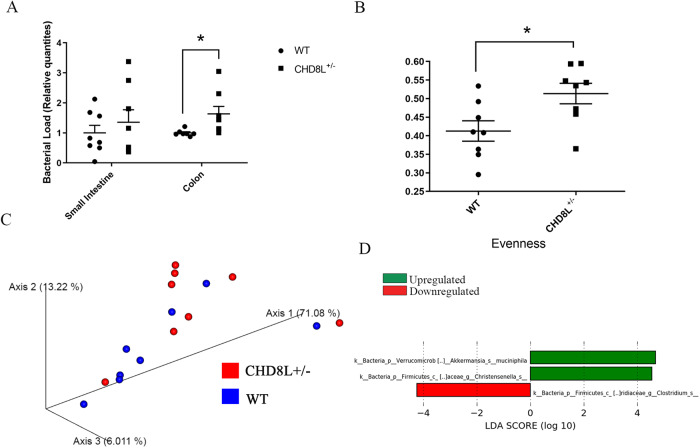
Analysis of microbiome in *Chd8L*^+/−^ mice. **A** Relative bacterial load in the small intestine and colon of WT and *Chd8L*^+/−^ mice (*p < 0.05, unpaired two-tailed t-test; *n* = 8/group). Shown are means ± SEM. **B** Measures of alpha diversity revealed significantly increased evenness in *Chd8L*^+/−^ mice relative to WT mice (*p < 0.05, Kruskal-Wallis pairwise analysis, *n* = 8/group). **C** Weighted UniFrac-based Principle Coordinates Analysis plot-based visualization of the microbial communities in *Chd8L*^+/−^ and WT mice. **D** Bacterial taxa that were overrepresented (red) or underrepresented (green) in *Chd8L*^+/−^ mice, as identified according to LefSe analyses of 16 S high-throughput sequencing data.

Given the potential role of the microbiota in autism and the increased antimicrobial peptide expression observed in *Chd8L*^*+/−*^ mice, 16 S rDNA sequencing was performed to compare the composition of the fecal microbiome between *Chd8L*^*+/−*^ and WT controls at 8 weeks of age. A significant increase in overall bacterial load (Fig. 3A) and alpha diversity (Fig. 3B) was measured in the colon of *Chd8L*^+/−^ mice, while no corresponding differences in beta diversity were evident between these genotypes (Fig. 3C). LEfSe analyses of the 16 S sequencing data revealed differential abundance of three bacterial taxa in the two genotypes (Fig. 3D). Notably, *Chd8L*^*+/−*^ mice exhibited increased abundance of *Akkermansia muciniphila (A. muciniphila)*, which has been widely implicated in the regulation of immune and neurological function.

### Generation of gut epithelial cell-specific *Chd8* haploinsufficient mice

**Fig. 4:**
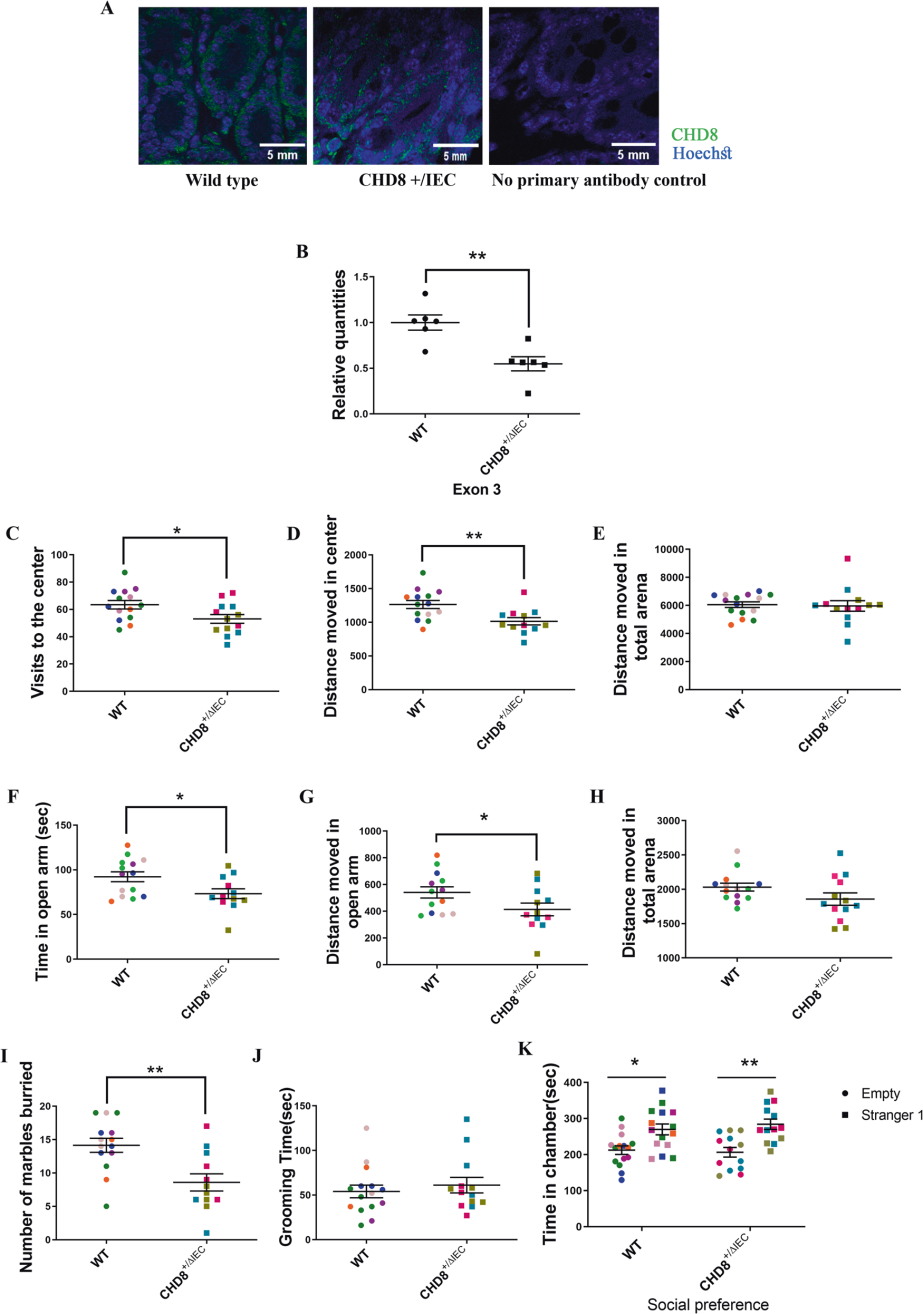
Behavioral analysis of gut epithelial-specific *CHD8* haploinsufficient mice. **A** Representative CHD8 staining of wild-type (WT) and *Chd8*^+/ΔIEC^ gut epithelial layer. Samples were stained for CHD8 (green), and Hoechst dye was used as a nuclear counterstain (blue). **B** Relative quantification of *Chd8* exon 3 was performed via real-time PCR (*p < 0.05, unpaired two-tailed t-test; *n* = 6 per genotype). **C**–**E** Open-field test results. **C** Visits to the center by *Chd8*^+/ΔIEC^ mice (*n* = 13) vs. WT controls (*n* = 14) (*p < 0.05, unpaired two-tailed t-test). **D** Distance moved in the center by *Chd8*^+/ΔIEC^ mice (*n* = 13) vs. WT controls (*n* = 10) (**p < 0.01, unpaired two-tailed t-test). **E** Distance moved in the arena (*p* = 0.8). **F**–**H** Elevated plus maze test results. **F** Time spent in the open arms of the maze (*p < 0.05, unpaired two-tailed t-test; WT: *n* = 14, *Chd8*^*+/*^^ΔIEC^: *n* = 11). **G** Distance moved in the open arms of the maze (*p < 0.05, unpaired two-tailed t-test; WT: *n* = 14, *Chd8*^*+/*^^ΔIEC^: *n* = 12). **H** Distance moved in the arena (*p* = 0.1). **I** Marble-burying activity by *Chd8*^+/ΔIEC^ vs. WT mice (**p < 0.01, unpaired two-tailed t-test; WT: *n* = 14, *Chd8*^*+/*^^ΔIEC^: *n* = 12). **J** Grooming time (*p* > 0.05). **K** Social preference test of WT and *Chd8*^+/ΔIEC^ mice for stranger mice. (*p < 0.05, two-way ANOVA with Tukey’s post-hoc test; WT: *n* = 15, *Chd8*^+/ΔIEC^: *n* = 13). Data are means ± SEM.

Given the complex gut phenotype of *Chd8L*^+/−^ mice, and to facilitate exploration of the role of gut dysfunction in autism-related behavior, *Chd8* was specifically knocked out of gut epithelial cells using a Cre-lox system, with the expression of Cre under the control of the gut epithelium-specific villin promoter. In all experiments, results were compared between *Chd8* gut epithelial haploinsufficient mice (Villin-Cre/Chd8flx^+/−^, now refered to as CHD8^+/ΔIEC^ mice) and WT littermate controls (Chd8flx^+/−^). The gene knockdown was validated by immunostaining of the gut of CHD8^+/ΔIEC^ and WT mice (Fig. 4A) and by RT-PCR (Fig. 4B). In the open field test, *Chd8*^+/ΔIEC^ mice exhibited fewer visits to the center (Fig. 4C) and decreased distance moved in the center (Fig. 4D), suggesting anxiety-like phenotypes, while total distance moved was comparable with that of WT controls (Fig. 4E). In the elevated plus maze test, *Chd8*^+/ΔIEC^ mice spent less time in the open arms of the maze (Fig. 4F) and moved less distance in the open arms (Fig. 4G), further indicating anxiety-like behavioral phenotypes. A reduction in marble-burying was also observed in *Chd8*^+/ΔIEC^ mice relative to WT controls (Fig. 4I). Grooming time was similar in the two mouse groups (Fig. 4J), as was performance in a three-chamber social behavior test (Fig. 4K), indicating that repetitive and social behavioral phenotypes, respectively, were unchanged in these mice. Cre expression alone had no impact on any of the tested behaviors (Supplementary Fig. 4). In summary, anxiety-like behaviors were specifically induced by the knockout of *Chd8* in the gut epithelial cells.

### *Chd8*^+/ΔIEC^ mice exhibit reduced tuft cell counts

**Fig. 5:**
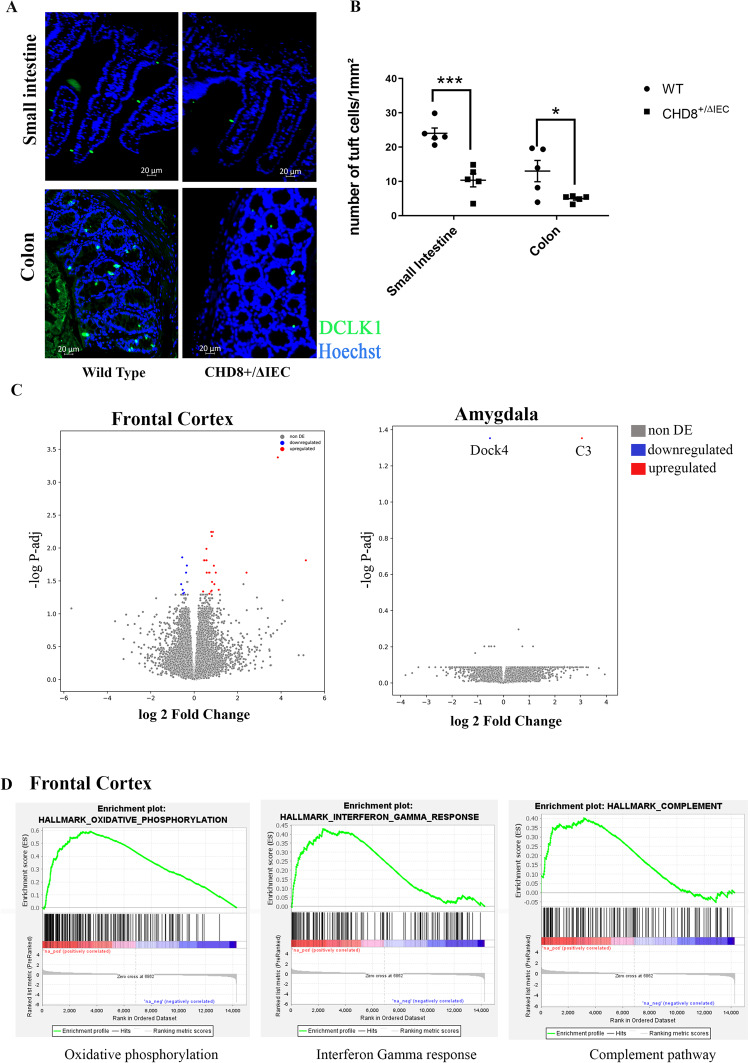
Morphological and transcriptional analysis of gut epithelial-specific *CHD8* haploinsufficient mice. **A**, **B** Representative DCLK1 staining of small intestine and colon samples (**A**), with corresponding quantification of the number of tuft cells per mm^2^ (**B**) in these tissue compartments (*p < 0.05, ***p < 0.001; unpaired two-tailed t-test, *n* = 5/group). Data are means ± SEM. **C** Volcano plot highlighting differentially expressed gene distributions in *Chd8*^+/ΔIEC^ brain regions, including the frontal cortex and amygdala. Each point represents a genein *Chd8*^+/ΔIEC^ mice, plotted against the level of statistical significance (−log10 adjusted *p*-value) and fold-change in expression (log2 (*Chd8*^+/ΔIEC^ vs. WT). **D** Gene Set Enrichment Analysis indicating gene pathways enriched in the frontal cortex, as determined by the fold-change difference in gene expression between the WT and *Chd8*^+/ΔIEC^ groups.

In light of the decreases in tuft cell numbers measured in mice exhibiting systemic *Chd8* haploinsufficiency, immunohistochemical staining was performed to detect tuft cells in the gastrointestinal epithelium of *Chd8*^+/ΔIEC^ mice. A significant reduction in the number of DCLK1-positive tuft cells was evident in both the small intestine and colon of 8-week-old *Chd8*^+/ΔIEC^ mice (Fig. 5A, B), whereas Cre expression alone did not affect tuft cell counts (Supplementary Fig. 5). While gut-specific *Chd8* haploinsufficiency was sufficient to induce a drop in tuft cell numbers in the gut epithelium, no corresponding differences between *Chd8*^+/ΔIEC^ and WT mice were observed with respect to the length of the colon, small intestine, or whole gut (Supplementary Fig. 3B). The bacterial load in collected stool samples was also comparable between WT and *Chd8*^+/ΔIEC^ mice (Supplementary Fig. 3A).

### Transcriptome analyses of the brains of *Chd8*^+/ΔIEC^ mice

To gain further insight into the molecular mechanisms possibly underlying the increased anxiety phenotypes of *Chd8*^+/ΔIEC^ mice, transcriptomic analyses were performed using frontal cortex and amygdala samples collected from *Chd8*^+/ΔIEC^ and WT control mice. In total, 35 genes in the frontal cortex and 2 genes in the amygdala were differentially expressed (FDR < 0.05) in *Chd8*^+/ΔIEC^ mice relative to the corresponding tissues from WT animals (Fig. 5C). Interestingly, levels of complement subunit 3 (C3), an antimicrobial factor that has been implicated in anxiety [26], were upregulated in the amygdala of *Chd8*^+/ΔIEC^ mice. A gene set enrichment analysis (GSEA) of the genes differentially expressed in the frontal cortex also identified upregulation of the complement pathway, as well as the oxidative phosphorylation and interferon-gamma response pathways (Fig. 5D).

### Antibiotic treatment attenuates social behavior deficits in *Chd8L*^+/−^ mice

**Fig. 6:**
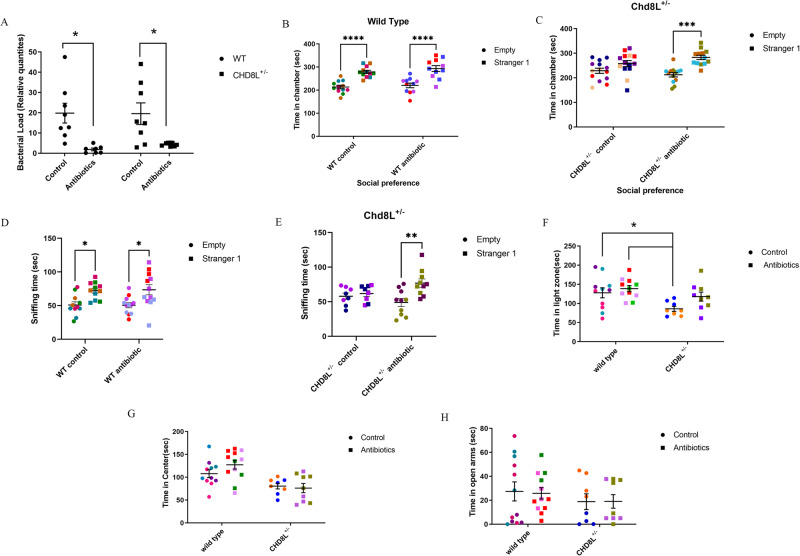
Antibiotic treatment of *CHD8L* + */*– mice. **A** Real-time PCR-based determination of bacterial load following antibiotic treatment of WT mice relative to untreated WT controls and *Chd8L*^+/−^ mice relative to untreated *Chd8L*^+/−^ controls (*p < 0.05, two-way ANOVA with Tukey’s post-hoc test; *n* = 8/group). **B**, **C** Social preference tests. **B** WT control (*n* = 12) and antibiotic-treated WT (*n* = 11) mouse preference for stranger mice (two-way ANOVA with Tukey’s post-hoc test). **C** *Chd8L*^+/−^ control mouse (*n* = 14) and antibiotic-treated *Chd8L*^+/−^ mouse (*n* = 13) preference for stranger mice (two-way ANOVA with Tukey’s post-hoc test). **D** WT control (*n* = 11) and antibiotic-treated WT (*n* = 12) mouse sniffing of stranger mice (two-way ANOVA with Tukey’s post-hoc test). **E** Time spent by *Chd8L*^+/−^ control (*n* = 8) and antibiotic-treated *Chd8L*^+/−^ (*n* = 10) mice sniffing empty chamber or stranger mice (two-way ANOVA with Tukey’s post-hoc test). **F** Dark light test of *Chd8L*^+/−^control mice (*n* = 8) (*p < 0.05, two-way ANOVA with Tukey’s post-hoc test; antibiotic-treated *Chd8L*^+/−^ mice: *n* = 9.) **G** Time spent in the center in an open field test. **H** Time spent in the open arms in an elevated plus maze test. Data are presented as means ± SEM.

Considering the increased bacterial load detected in *Chd8L*^+/−^ mice, the impact of antibiotics on autism-like phenotypes was evaluated. A 3-week treatment of 5-week-old WT and *Chd8L*^+/−^ mice with ciprofloxacin (0.04 g/L), metronidazole (0.2 g/L) and vancomycin (0.1 g/L), resulted in significantly reduced bacterial loads in the stool of the mice of both groups relative to untreated animals (Fig. 6A). In a three-chamber social interaction test, WT mice exhibited a preference for stranger mice over the empty cage, irrespective of antibiotic treatment (Fig. 6B), as measured by the time spent in each chamber. In contrast, while untreated *Chd8L*^+/−^ mice did not exhibit any preference for stranger mice, consistent with impaired social behaviors, this deficit was attenuated following antibiotic treatment (Fig. 6C). WT animals with or without antibiotic treatment, also spent more time sniffing stranger mice relative to the empty chamber (Fig. 6D). In contrast, untreated *Chd8L*^+/−^ mice spent similar periods of time sniffing the empty chamber and unfamiliar mice, while antibiotic treatment led them to spend significantly more time sniffing the unfamiliar mice relative to the empty chamber (Fig. 6E). In a dark-light test, *Chd8L*^+/−^ mice spent less time in the open arms relative to WT controls, a difference that was eliminated by antibiotic treatment (Fig. 6F). In open-field (Fig. 6G) and elevated plus maze tests, the mice of both groups exhibited similar behaviors (Fig. 6H). Taken together, antibiotic treatment reversed some of the phenotypic changes evident in *Chd8L*^+/−^ mice, with a particularly strong impact on social and anxiety phenotypes.

## Discussion

An association between GI symptoms and autism has long been documented, with a reported increase in the odds of developing GI symptoms with heightened autism severity [4]. Individuals harboring *CHD8* mutations also frequently exhibit a range of GI symptoms [15]. The present study sought to explore the relationship between *CHD8* haploinsufficiency and GI dysfunction in mice, while also exploring the potential link between these factors and autism-related behavioral phenotypes. *Chd8L*^+/−^ mice exhibited increased intestinal permeability, consistent with that observed in patients with autism [12]. The mucus layer is, in part, responsible for maintaining the integrity of the intestinal barrier [27, 28]. We found that the mucus layer thickness in the colon and goblet cells counts in the small intestine were reduced in 8-week-old *Chd8L*^+/−^ mice. Given that goblet cells produce mucus [27], the decrease in goblet cell numbers likely reduced the mucus layer thickness, contributing to higher intestinal permeability. However, reduction in goblet cell numbers was only evident in the small intestine whereas the thinning of the mucus layer was observed in the colon. While it is not clear as to whether a decline in the goblet cell population in the small intestine can directly influence mucus in the colon, it is possible that mucus levels in the small intestine are also reduced in these animals, although this is not technically possible to measure.The impact of the goblet cell population in the small intestine on mucus levels in the colon, remains to be determined.

*Chd8L*^+/−^ mice also exhibited a higher bacterial load and greater alpha diversity in the colon. Transcriptomic and RT-PCR analyses measured high Reg3β and Reg3γ expression in the gut epithelial cells of *Chd8L*^+/−^ mice, which may, in part, serve to compensate for the higher bacterial load in these mice. While the functional consequences of the increased alpha diversity are not clear, the increased microbiome richness may be related to the overall increase in bacterial load. The observed overall increase in bacterial load was somewhat novel, in part because most microbiome research primarily assesses microflora diversity via 16 S sequencing without any corresponding quantification of bacterial load.

While few changes in bacterial taxa were abundant when comparing murine genotypes, an increase in *A. muciniphila* was observed in *Chd8L*^+/−^ mice. Notably, *A. muciniphila* has previously been proposed as a possible probiotic by impacting intestinal permeability [29], although it can also reportedly cause colitis in some settings. Multiple studies have also detected elevated *A. muciniphila* levels in the context of neurological conditions, including Parkinson’s disease, multiple sclerosis, and autism spectrum disorders [30]. Further research is thus warranted to understand the precise link between this bacterial species and neurological pathogenesis.

Transcriptomic analyses of the gut epithelial cells identified upregulation of several immune system-related genes in *Chd8L*^+/−^ as compared with WT mice, whereas tuft cell marker genes were downregulated. A prior RNA-seq analysis of the brains of adult mice of the same strain detected just five differentially expressed genes [23], whereas the present work found over 900 differentially expressed genes in the gut. This suggests that CHD8 has particularly important gut-specific functions during adulthood. Tuft cells induce type 2 immune responses and are a source of IL-25, which drives a feed-forward signaling pathway involving tuft cells and type 2 innate lymphoid cells (ILC2s) [31]. ILC2s serve as a source of IL-5, IL-9 and IL-13, which contribute to type 2 inflammation. IL-13 has also been shown to drive an increase in goblet cell numbers [31]. Reductions in tuft cell numbers may thereby contribute to a concomitant decrease in goblet cell abundance, thereby reducing the thickness of the mucus layer.

One of the most distinct behavioral phenotypes of *Chd8L*^+/−^ mice, first reported by Katayama et al., is an increase in anxiety-related behaviors [23]. Strikingly, specific deletion of *Chd8* from gut epithelial cells mice was sufficient to recapitulate the anxiety-like behaviors of *Chd8L*^+/−^ mice carrying systemic *Chd8L* haploinsufficiency. However, CHD8^+/ΔIEC^ mice did not exhibit any differences in bacterial load. Since approximately 30% of individuals with *CHD8* mutations display increased anxiety, these data suggest that this phenotype is tightly linked to gut dysregulation [15]. Social behavior deficits may be driven by other cell populations, such as gut immune cells or the enteric nervous system, or by the altered bacterial load, as supported by the effects of antibiotic treatment on social behavior in *Chd8L*^+/−^ mice.

In contrast to findings from *Chd8L*^+/−^ mice, in which tuft cell numbers were only reduced in the small intestine but not the colon, *Chd8*^+/ΔIEC^ mice exhibited fewer tuft cells in both the colon and small intestine relative to WT controls. Given that tuft cell numbers are highly regulated by local microenvironmental conditions, including immune cells, it is possible that systemic CHD8 deficiency masked the effects of epithelial cell *Chd8* haploinsufficiency on colonic tuft cell counts.

RNA-seq analysis of samples collected from both the frontal cortex and amygdala of *Chd8*^+/ΔIEC^ mice found significantly increased *C3* levels and decreased *Dock4* expression in the amygdala. Earlier studies have reported on the role of C3 in innate immunity as well as in anxiety-like behaviors. More specifically, C3-knockout mice exhibited reduced anxiety [32], and Alzheimer’s disease model mice experienced a drop in anxiety when treated with complement inhibitors [33]. In parallel, *Dock4* has been linked to the risk of autism, where *Dock4*-knockout mice reportedly exhibit autism-related behaviors [34]. *Dock4* has also been demonstrated a regulator of goblet cell differentiation and MUC2 production [35]. The dysregulation of immune-related factors, in the form of reduced tuft cell counts and increased expression of immune-related genes in the gastrointestinal tract of *Chd8L*^+/−^ mice, may link between gut-localized alterations and behavioral alterations. Alternatively, an increase in gastrointestinal permeability may allow entry of anxiogenic metabolites to the bloodstream and thereby reach the brain. The anxiety-like phenotypes in *Chd8L*^+/−^ mice may also be the result of pain or discomfort caused by changes in autonomic function [36, 37]. Possible sex-specific differences in the effects of Chd8L deficiencies remain to be determined.

Several works have associated distinct gut microbiome profiles with autism-related behaviors [6]. For example in a three-chamber sociability test, germ-free (GF) mice spent more time in an empty chamber than in a chamber containing stranger mice relative to control animals [6]. In a seperate study using the social novelty paradigm, GF mice spent more time with novel mice than familiar mice when compared with controls [38]. In a murine model of autism, administration of *Bacteroides fragilis*, reportedly increased social communication in the form of vocalizations, reduce anxiety and attenuate repetitive behaviors, although it had no impact on behavior in a three-chamber social interaction test [39]. Microbial transfer has also been found to alleviate certain autism-related symptoms in individuals with autism [40].

Antibiotic treatment was associated with abrogation of social behavior deficits in *Chd8L*^+/−^ mice, providing further support for the suggested benefit of antibiotic treatment in a subset of individuals with autism. For instance, Sandler et al. found that patients affected by regressive onset of autism, as defined by normal development up to two years of age, followed by the onset of autism-associated behaviors, exhibited short-term improvements upon oral vancomycin treatment [41]. In another study, children diagnosed with autism displayed improved SCERTS Assessment Process Observation (SAP-O) scores six months after treatment with antibiotics including amoxicillin and zithromycin [42]. Additional research will be needed to determine whether antibiotic treatment can relieve autism symptoms. Given that the underlying causes of autism can vary substantially across individuals, further research should focus on responses of subgroups of individuals with autism of specific genetic backgrounds or with specific behavioral phenotypes.

In conclusion, *Chd8L* haploinsufficiency induced changes in GI morphology, tuft cell counts, intestinal permeability, bacterial load and alpha diversity in mice. Specifically, *Chd8* knockout in gut epithelial cells (CHD8^+/ΔIEC^) caused anxiety-like behaviors, without impacting social behavior. Treatment of *Chd8L*^+/−^ mice with antibiotics rescued abnormal social behaviors. These results suggest that GI abnormalities may play a role in the symptomology and behavioral phenotypes of autism.

## Materials and Methods

### Animal models

All mice were bred and maintained in the animal facility of the Faculty of Medicine, Bar Ilan University, and experimental procedures were approved by Institute Animal Ethical Care and Use Committee (protocol number 16–8–2017). All mice are kept in a vivarium at 22 °C, on a 12 h light/dark cycle, with food and water available ad libitum. The C57BL/6 CHD8L ^+/−^ mice were as described by Katayama et al. [23], and have been previously phenotyped. The mice were kindly provided by Keiichi I. Nakayma, through Riken BRC mouse resource (Wako, Japan). C57BL/6 Chd8L^+/−^ male mice were crossed with female WT mice to produce WT and C57BL/6 Chd8L^+/−^ mice. To generate gut epithelial cell-specific CHD8 haploinsufficient mice, male villin-Cre^+/−^ mice were crossed with female CHD8fl/fl mice. All offspring were CHD8fl/wt, and half the offspring were also villin-cre^+/−^ (villin cre-positive). Cre-negative (WT) and Cre-positive (CHD8 floxed heterozygote) littermate offsprings were used in all experiments. Haploinsufficient mice were referred to as Chd8^+/ΔIEC^. The floxed CHD8 mice was purchased from Jackson Laboratories (stock number 031555). The test mice used in the experiments were always the first-generation offspring of the above-mentioned breeding schemes. Breeding was performed with the aim to produce 12 mice per experimental group. All behavioral tests were performed on 8–10-week-old mice. Only male mice were used throughout this study.

### Gut permeability assay

Fasting (6 h) 8-week-old mice were gavaged with 14 mL/kg body weight phosphate-buffered saline (PBS, pH 7.4) containing 22 mg/mL FITC-dextran (molecular mass 4.4 kDa; Sigma, St. Louis, MO, USA). A blood sample (150 µl) was collected by orbital retro bulbar puncture 2 h and 6 h after gavage. The blood samples were centrifuged (1000 X g at 4 °C), for 15 min, and the plasma supernatant (50 µl) was mixed with an equal volume of PBS (pH 7.4) and placed in the well of a 96-well microplate (black). The concentration of FITC-dextran was determined by spectrophotometry, at an excitation wavelength of 485 nm (20 nm band width) and an emission wavelength of 530 nm (25 nm band width).

### Histology preparation

The small intestine and colon were excised from 8-week-old mice, immediately submerged in Ethanol-Carnoy’s Fixative at 4 °C for 2 h, and then placed in 100% ethanol, and then in 50%, 75% and 100% xylene, before being embedded in paraffin. Sections (5 µm) were cut from the paraffin blocks.

### Alcian-blue periodic acid Schiff reagent (Ab/PAS) staining

Sections were deparaffinised and stained with alcian blue (Sigma, St. Louis, MO, USA; A5268) for 15 min. After washing with distilled water, they were treated with periodic acid (Sigma, St. Louis, MO, USA; p7875) for 5 min, washed in distilled water for 3 min, and then stained with Schiff reagent (Sigma St. Louis, MO, USA; 3952016) for 10 min. After rinsing sections under running tap water for 5 min, the nuclei were stained with haematoxylin for 1 min. Sections were then dipped into acid alcohol, dehydrated and mounted. Goblet cell counts and mucus layer thicknesswere determined using the ZEN Desk software.

### Immunostaining

After deparaffinisation, antigen was retrieved by incubating sections in sodium citrate buffer (pH = 6) for 20 min, at 60 °C. After cooling, sections were permeabilized with 0.1% Triton X-100, for 10 min and then blocked for 3 h, at room temperature, with 2% BSA (Calbiochem, San Diego, CA, USA; 126575) and 1% goat serum in 0.1% Tris buffer saline and triton-X (TbTx). The tissue sections were then immunofluorescently stained for 1 h, at room temperature and then overnight at 4 °C, with the following anitbodies: anti-CHD8 (1:100) (Abcam, Cambridge, UK; ab84527), anti-ZO-1 (1:200) (Thermo Fisher Scientific; 40–2200) and anti-DCLK1 (1:200) (Abcam, Cambridge, UK; ab31704). The sections were then washed three times with cold TbTx, for 10 min each, and incubated with Alexa 488-labeled anti-rabbit secondary antibody (1:200) (Jackson Immune Research Laboratories, West Grove, PA; 111–545–144) at room temperature for 2 h. After washing three times, for 10 min each, with TbTx, sections were stained with Hoechst (Sigma, St. Louis, MO, USA) (1:1000) and then mounted. The sections were examined with a fluorescence microscope.

### Stool collection, DNA extraction and sequencing of 16 s rRNA gene

Mouse fecal samples were collected directly from the small intestine and colon and stored at −80 °C until analyses. DNA was isolated using the PureLink^TM^ Microbiome DNA Purification Kit, according to the manufacturer’s instructions. The V4 region of the bacterial 16 S rRNA gene was PCR-amplified using the 515 F and 806 R primers [http://www.earthmicrobiome.org/]. Forward primers included unique 12-base barcodes in order to tag PCR products. The PCR reaction included PrimeSTAR Max Premix 1x (Takara Bio, Shiga, JP), 0.4 *μ*M of each primer and 30–100 ng DNA template. Reaction conditions were as follows: initial denaturing for 3 min at 95 °C, followed by 30 cycles of 10 s at 95 °C, 5 s at 55 °C and 5 s at 72 °C. PCR reactions were performed in duplicates for each sample, pooled and purified using the Agencourt AMPure XP kit (Beckman Coulter, Brea, CA, USA). Purified PCR products were quantified using a Qubit dsDNA HS assay kit (Life Technologies, Carlesbad, CA, USA) and 50 ng of each sample was pooled for further sequencing on the Illumina MiSeq platform.

### Bioinformatic analyses of 16 S rRNA gene sequences

16 s rRNA sequencing data were analyzed by the QIIME 1 pipeline [43]. Alpha diversity (within-community diversity) was estimated by the Gini coefficient, a measure of community evenness. Beta diversity (between-community diversity) was calculated using weighted UniFrac distances. The diversity parameters were compared between groups using a nonparametric t-test with Monte Carlo permutations (999) to calculate *p* values. The Benjamini and Hochberg FDR method was then used to correct *p* values for multiple comparisons between different pairs of groups. Linear discriminant analysis (LDA) effect size (LefSe) was determined to identify differences in relative abundance at different taxonomic levels. Values used in analysis were alpha = 0.05 and LDA threshold of 2.0.

### Gut epithelial cell isolation

Epithelial cells were collected from 5 wild type and 7 Chd8L^+/−^ mice. All mice were 8-week-old male littermates. Epithelial cells were isolated as previously described by Zeineldin et al. [44]. Intestinal sections were washed with PBS, and then treated with 0.04% sodium hypochloride for 15 min, on ice. Specimens were then incubated in solution B (2.7 mM KCl, 150 mM NaCl, 1.2 mM KH2PO4, 680 mM Na2HPO4, 1.5 mM EDTA, 0.5 mM DTT) for 15 min, on ice. The specimens were then transferred to PBS and vortexed for 50 s. After repeating tincubation and vortex cycles three times, the solution was centrifuged at 1000 g, 10 min, 4 °C. Epithelial cells were concentrated in the pellet. The collected pellet were resuspended in buffer RLT for RNA purification using the RNeasy Micro kit (Qiagen, Hilden, DE; 74004), following the standard protocol with on-column DNase digestion.

### RNA extraction from brain regions

Mice were sacrificed by rapid decapitation and brains were quickly removed. Sections (2 mm) of the frontal cortex and amygdala were isolated using a 13 G needle, and immediately frozen on dry ice. Total RNA was extracted using the RNeasy Mini Kit (Qiagen, Hilden, DE), according to the manufacturer’s protocol.

### RNA sequencing and analysis

Sequencing libraries were prepared using the NEBNext Poly(A) mRNA Magnetic Isolation Module & NEBNext® Ultra™ II RNA Library Prep Kit for Illumina® sequencing. The 75-base-pair single-end sequencing was performed on the Nextseq 75SR. Reads were mapped to the *Mus musculus* reference genome (mm10) using the Tophat2 software (release Tophat2.0.12). Genes differentially expressed in gut epithelial cells are listed in Supplementary table 1 and mapped reads can be found in Supplementary Table 2. Differentially expressed genes and mapped read files for frontal cortex and amygdala RNA-seq is found in supplementary table 6. Differential gene expression analysis was performed using the DESeq2 pipeline. Enrichment analyses for the Gene Ontology (GO) terms were performed using the online ToppGene Suite software. GO terms were considered significant when the Benjamini and Hochberg FDR adjusted *p* value was below 0.05. The raw data and read count data from this analysis are available at GSE182815. GSEA was performed on genes from the RNA-seq analysis that were ranked by fold-change in expression between the two experimental groups. GSEA was performed as described previously [45], using GSEA v.2.0.1 (http://www.broadinstitute.org/gsea).

### Real-time PCR

RNA was converted to cDNA using the Maxima H minus first strand cDNA synthesis kit with dsDNAse (Thermo Scientific, Waltham, MA, USA). Real-time PCR for tuft cell markers, antimicrobial peptide genes, CHD8 exon 1, exon 11–13 and exon 3 was performed using the Fast Start Universal SYBR Green Master (Roche, Basel CH) and ViiA™7 Real-Time PCR System (Life Technologies, Carlesbad, CA, USA). The PCR program was run for 40 cycles, and included a melting temperature of 95 °C for 10 s, and an annealing temperature of 60 °C for 30 s. Relative quantification of tuft cell abundance in the gut was performed using the ΔΔCt method. The primers used in the reactions are listed in Supplementary Table 4.

### Bacterial load quantification

Bacterial load was quantified as described previously by Nadkarni et al. [46], using Taqman and the ViiA™7 Real-Time PCR System (Life Technologies, Carlesbad, CA, USA). The PCR thermocycler settings were as follows: 40 cycles of 95 °C for 20 s and 60 °C for 20 s. The ΔΔCt method was used to quantify relative bacterial abundance. Primers and probes used in these analyses are listed in Supplementary Table 4.

#### Antibiotic Treatment

Chd8L^+/−^ mice and their WT controls (5 weeks old) were administered a combination of ciprofloxacin (0.04 g/L), metronidazole (0.2 g/L) and vancomycin (0.1 g/L) via their drinking water, for 3 weeks. All antibiotics were purchased from Sigma (St. Louis, MO, USA).

#### Behavioral testing

Mice were habituated to the room for at least 1 h before commencement of each test. Each test was performed on a separate day, usually with a 1-day interval between each test. Animal movement was filmed with a camera, and animal behaviour was tracked and automatically scored by the Noldus Software “ethovision”. All tested animals were included in the analysis unless they left the experimental arena. When necessary (e.g., antibiotics vs. no antibiotics), animals of the same genotype were randomly assigned to different experimental groups.

#### Open-field test

The mouse is placed in the corner of a plastic square box (50 × 50 cm) where it is allowed to move freely for 10 min, under ~120LUX of light. During this time, a camera films and tracks the behavior of the animal, including distance traveled.

#### Light/dark box test

The mouse is placed in a dark plastic chamber (75 × 75 cm) with an opening to a highly lit chamber (~1200 lux). The mouse is allowed to freely move between the two chambers for 5 min. During this time, a camera films and tracks the behavior of the animal, including its exact location inside the box, velocity, and distance travelled.

#### Elevated plus maze

The mouse is placed in the center of a four arms maze. Each arm is 30 cm in length, and two are closed and two are open. The maze is approximately one meter high. The mouse is free to choose which arm it enters for a 5 min period. During this time, a camera films and tracks the behavior of the animals, including where they are found in the maze, velocity, and distance travelled.

#### Social interaction test

The test is performed in a non-glare Perspex box (60 × 40 cm) with two partitions that divide the box into three chambers, left, center and right (20 × 40 cm each). The mouse is placed in the middle chamber for habituation (5 min) while the entry to both side chambers is barred. The test mouse was then allowed to explore the entire arena (10 min), and to freely choose between interacting with a novel mouse in one chamber or remaining in an empty chamber (social test). During this time, a camera films and tracks the behavior of the animals, and time spent in each chamber is measured. To assess mouse sniffing, the interaction between the nose of test mouse and the nose or body of a stranger mouse was analyzed. Interaction within an area of 2 mm was defined as positive sniffing interaction.

#### Marble-burying test

Repetitive marble burying was measured in a non-glare Perspex (20 × 40 cm) chamber. Twenty green glass marbles (15 mm diameter) were arranged in a 4 × 5 grid that covered 2/3 of the apparatus on top of 5 cm clean bedding. Each mouse was placed in the corner that did not contain the marbles and was given 30 min to explore the chamber, after which the number of marbles buried, was counted. “Buried” was defined as 2/3 covered by bedding. Testing was performed under dim light (25 lux).

#### Self-grooming

Each mouse is placed individually into an open-field chamber. After a 10 min habituation period, each mouse is scored using parameters of cumulative time spent grooming during a 20 min session.

#### Statistics

A two-tailed unpaired T-test or two way ANOVA was performed using Graphpad prism 9.3 software. Data are presented as mean ± standard error of the mean (SEM). Supplementary Table 5 summarizes the results of all statistical tests mentioned in this manuscript.

### Supplementary information

Supplementary Figures

Supplementary table 1

Supplementary table 2

Supplementary table 3

Supplementary table 4

Supplementary table 5

Supplementary table 6

## References

[1] Xu Q, Liu YY, Wang X, Tan GH, Li HP, Hulbert SW (2018). Autism-associated CHD8 deficiency impairs axon development and migration of cortical neurons. Mol Autism.

[2] Jolanta Wasilewska J, Klukowski M (2015). Gastrointestinal symptoms and autism spectrum disorder: links and risks – a possible new overlap syndrome. Pediatr Heal Med Ther.

[3] Buie T, Campbell DB, Fuchs GJ, Furuta GT, Levy J, Vandewater J (2010). Evaluation, diagnosis, and treatment of gastrointestinal disorders in individuals with ASDs: a consensus report. Pediatrics.

[4] Mayer EA, Padua D, Tillisch K (2014). Altered brain-gut axis in autism: comorbidity or causative mechanisms?. Bioessays.

[5] Wang LW, Tancredi DJ, Thomas DW (2011). The prevalence of gastrointestinal problems in children across the United States with autism spectrum disorders from families with multiple affected members. J Dev Behav Pediatr.

[6] Oh D, Cheon KA (2020). Alteration of gut microbiota in autism spectrum disorder: An overview. J Korean Acad Child Adolesc Psychiatry..

[7] Strati F, Cavalieri D, Albanese D, De Felice C, Donati C, Hayek J (2017). New evidences on the altered gut microbiota in autism spectrum disorders. Microbiome.

[8] Rose DR, Yang H, Serena G, Sturgeon C, Ma B, Careaga M (2018). Differential immune responses and microbiota profiles in children with autism spectrum disorders and co-morbid gastrointestinal symptoms. Brain Behav Immun.

[9] Kang DW, Ilhan ZE, Isern NG, Hoyt DW, Howsmon DP, Shaffer M (2018). Differences in fecal microbial metabolites and microbiota of children with autism spectrum disorders. Anaerobe.

[10] Sauer AK, Bockmann J, Steinestel K, Boeckers TM, Grabrucker AM (2019). Altered intestinal morphology and microbiota composition in the autism spectrum disorders associated SHANK3 mouse model. Int J Mol Sci.

[11] D’Eufemia P, Celli M, Finocchiaro R, Pacifico L, Viozzi L, Zaccagnini M, et al. Abnormal intestinal permeability in children with autism. Acta Paediatr Int J Paediatr. 1996; 85. 10.1111/j.1651-2227.1996.tb14220.x. 10.1111/j.1651-2227.1996.tb14220.x 8888921

[12] De Magistris L, Familiari V, Pascotto A, Sapone A, Frolli A, Iardino P (2010). Alterations of the intestinal barrier in patients with autism spectrum disorders and in their first-degree relatives. J Pediatr Gastroenterol Nutr.

[13] Ma J, Rubin BK, Voynow JA (2018). Mucins, mucus, and goblet cells. Chest.

[14] O’Roak BJ, Vives L, Girirajan S, Karakoc E, Krumm N, Coe BP (2012). Sporadic autism exomes reveal a highly interconnected protein network of de novo mutations. Nature.

[15] Bernier R, Golzio C, Xiong B, Stessman HA, Coe BP, Penn O (2014). Disruptive CHD8 mutations define a subtype of autism early in development. Cell.

[16] Stessman HAF, Xiong B, Coe BP, Wang T, Hoekzema K, Fenckova M (2017). Targeted sequencing identifies 91 neurodevelopmental-disorder risk genes with autism and developmental-disability biases. Nat Genet.

[17] Iossifov I, O’Roak BJ, Sanders SJ, Ronemus M, Krumm N, Levy D (2014). The contribution of de novo coding mutations to autism spectrum disorder. Nature.

[18] Stolerman ES, Smith B, Chaubey A, Jones JR (2016). CHD8 intragenic deletion associated with autism spectrum disorder. Eur J Med Genet.

[19] Marfella CG, Imbalzano AN (2007). The Chd family of chromatin remodelers. Mutat Res.

[20] Nishiyama M, Skoultchi AI, Nakayama KI (2012). Histone H1 recruitment by CHD8 is essential for suppression of the Wnt–β-Catenin signaling pathway. Mol Cell Biol.

[21] Durak O, Gao F, Kaeser-Woo YJ, Rueda R, Martorell AJ, Nott A (2016). Chd8 mediates cortical neurogenesis via transcriptional regulation of cell cycle and Wnt signaling. Nat Neurosci.

[22] Noelanders R, Vleminckx K (2017). How Wnt signaling builds the brain: bridging development and disease. Neuroscientist.

[23] Katayama Y, Nishiyama M, Shoji H, Ohkawa Y, Kawamura A, Sato T (2016). CHD8 haploinsufficiency results in autistic-like phenotypes in mice. Nature.

[24] Ting H-A, von Moltke J (2019). The immune function of tuft cells at gut mucosal surfaces and beyond. J Immunol.

[25] Haber AL, Biton M, Rogel N, Herbst RH, Shekhar K, Smillie C (2017). A single-cell survey of the small intestinal epithelium. Nature.

[26] Westacott LJ, Humby T, Haan N, Brain SA, Bush EL, Toneva M, et al. Complement C3 and C3aR mediate different aspects of emotional behaviours; relevance to risk for psychiatric disorder. Brain Behav Immun. 2022; 99. 10.1016/j.bbi.2021.09.005. 10.1016/j.bbi.2021.09.005 34543680

[27] Herath M, Hosie S, Bornstein JC, Franks AE, Hill-Yardin EL (2020). The role of the gastrointestinal mucus system in intestinal homeostasis: implications for neurological disorders. Front Cell Infect Microbiol..

[28] Johansson MEV, Ambort D, Pelaseyed T, Schütte A, Gustafsson JK, Ermund A (2011). Composition and functional role of the mucus layers in the intestine. Cell Mol Life Sci..

[29] Luo Y, Lan C, Li H, Ouyang Q, Kong F, Wu A (2022). Rational consideration of Akkermansia muciniphila targeting intestinal health: advantages and challenges. npj Biofilms Microbiomes.

[30] Cirstea M, Radisavljevic N, Finlay BB (2018). Good bug, bad bug: Breaking through microbial stereotypes. Cell Host Microbe.

[31] Von Moltke J, Ji M, Liang HE, Locksley RM (2016). Tuft-cell-derived IL-25 regulates an intestinal ILC2-epithelial response circuit. Nature.

[32] Shi Q, Colodner KJ, Matousek SB, Merry K, Hong S, Kenison JE (2015). Complement C3-deficient mice fail to display age-related hippocampal decline. J Neurosci.

[33] P. Kulkarni A, A. Govender D, J. Kotwal G, A. Kellaway L (2011). Modulation of anxiety behavior by intranasally administered vaccinia virus complement control protein and curcumin in a mouse model of Alzheimers disease. Curr Alzheimer Res.

[34] Guo D, Peng Y, Wang L, Sun X, Wang X, Liang C (2021). Autism-like social deficit generated by Dock4 deficiency is rescued by restoration of Rac1 activity and NMDA receptor function. Mol Psychiatry.

[35] Qin T, Yang J, Huang D, Zhang Z, Huang Y, Chen H (2021). DOCK4 stimulates MUC2 production through its effect on goblet cell differentiation. J Cell Physiol.

[36] Koloski NA, Jones M, Talley NJ (2012). Investigating the directionality of the brain-gut mechanism in functional gastrointestinal disorders. Gut.

[37] Stasi C, Rosselli M, Bellini M, Laffi G, Milani S. Altered neuro-endocrine-immune pathways in the irritable bowel syndrome: The top-down and the bottom-up model. J Gastroenterol. 2012; 47. 10.1007/s00535-012-0627-7. 10.1007/s00535-012-0627-7 22766747

[38] Arentsen T, Raith H, Qian Y, Forssberg H, Heijtz RD (2015). Host microbiota modulates development of social preference in mice. Microb Ecol Heal Dis.

[39] Hsiao EY, McBride SW, Hsien S, Sharon G, Hyde ER, McCue T (2013). Microbiota modulate behavioral and physiological abnormalities associated with neurodevelopmental disorders. Cell.

[40] Kang DW, Adams JB, Coleman DM, Pollard EL, Maldonado J, McDonough-Means S (2019). Long-term benefit of Microbiota Transfer Therapy on autism symptoms and gut microbiota. Sci Rep.

[41] Sandler RH, Finegold SM, Bolte ER, Buchanan CP, Maxwell AP, Väisänen ML (2000). Short-term benefit from oral vancomycin treatment of regressive-onset autism. J Child Neurol.

[42] Kuhn M, Grave S, Bransfield R, Harris S (2012). Long-term antibiotic therapy may be an effective treatment for children co-morbid with Lyme disease and Autism Spectrum Disorder. Med Hypotheses.

[43] Caporaso JG, Kuczynski J, Stombaugh J, Bittinger K, Bushman FD, Costello EK (2010). QIIME allows analysis of high-throughput community sequencing data. Nat Methods.

[44] Zeineldin M, Neufeld K (2012). Isolation of epithelial cells from mouse gastrointestinal tract for western blot or RNA analysis. Bio-Protocol.

[45] Subramanian A, Tamayo P, Mootha VK, Mukherjee S, Ebert BL, Gillette MA (2005). Gene set enrichment analysis: A knowledge-based approach for interpreting genome-wide expression profiles. Proc Natl Acad Sci USA.

[46] Nadkarni MA, Martin FE, Jacques NA, Hunter N (2002). Determination of bacterial load by real-time PCR using a broad-range (universal) probe and primers set. Microbiology.

