# Supplementary material for: CHD8 regulates gut epithelial cell function and affects autism-related behaviors through the gut-brain axis
Source: Transl Psychiatry. 2023 Oct 2;13:305. doi: 10.1038/s41398-023-02611-2 (PMC10545671; doi:10.1038/s41398-023-02611-2)
Supplement: Supplementary file 1 — Supplementary Figures [file 41398_2023_2611_MOESM1_ESM.docx]

**Supplementary Figure 1.**

**
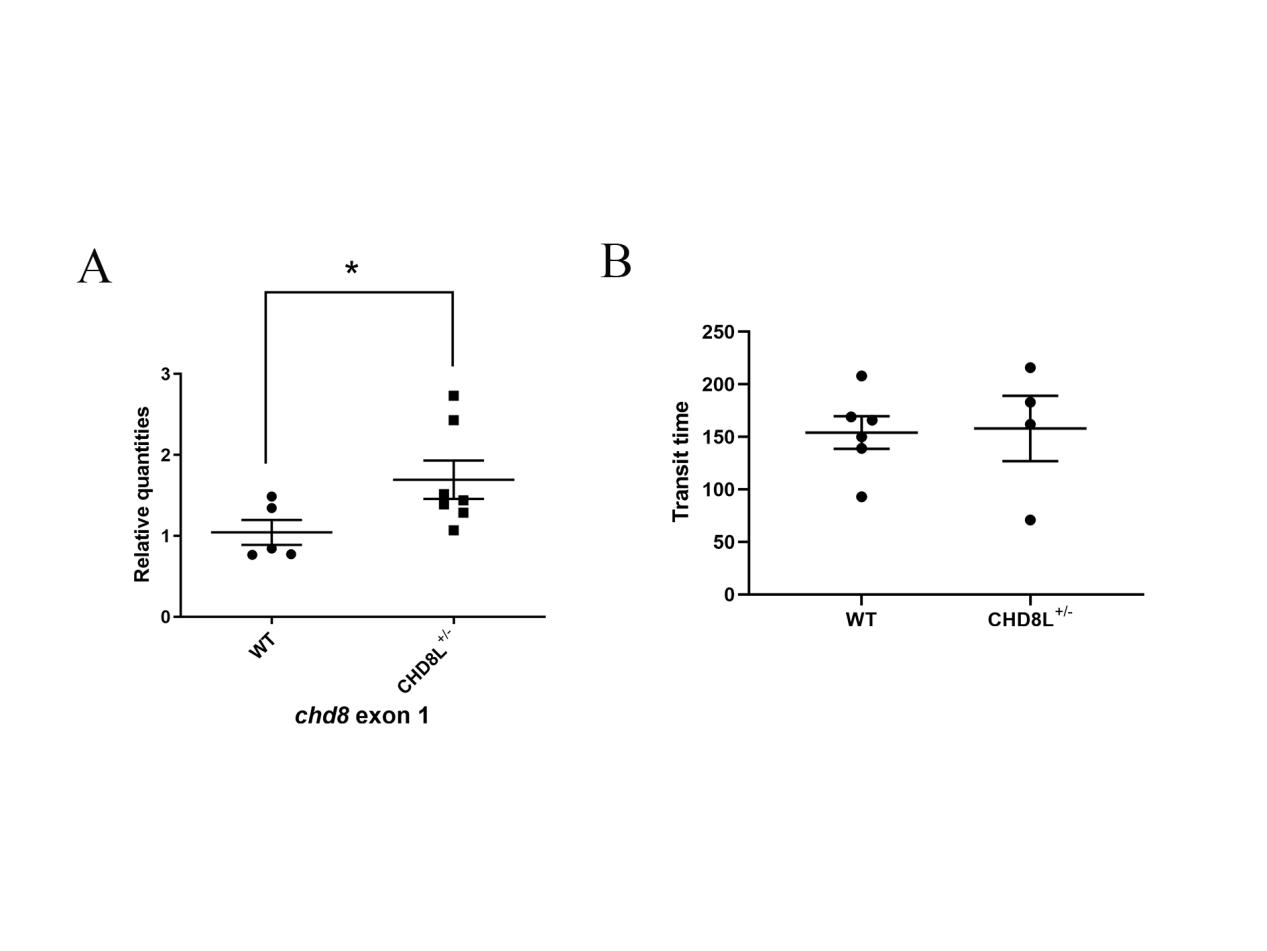
**

**A**. Real-time PCR analyses of *Chd8* exon 1. Relative *Chd8* expression was significantly increased in *Chd8L*^+/-^ mice (*p < 0.05, two-tailed unpaired t-test; WT: n=5, *Chd8L*^+/-^, n=7). **B**. Transit time assay (p > 0.05, two-tailed unpaired t-test; WT: n=6, *Chd8L*^+/-^, n=4). Data are presented as means ± SEM.

**Supplementary Figure 2.**

**
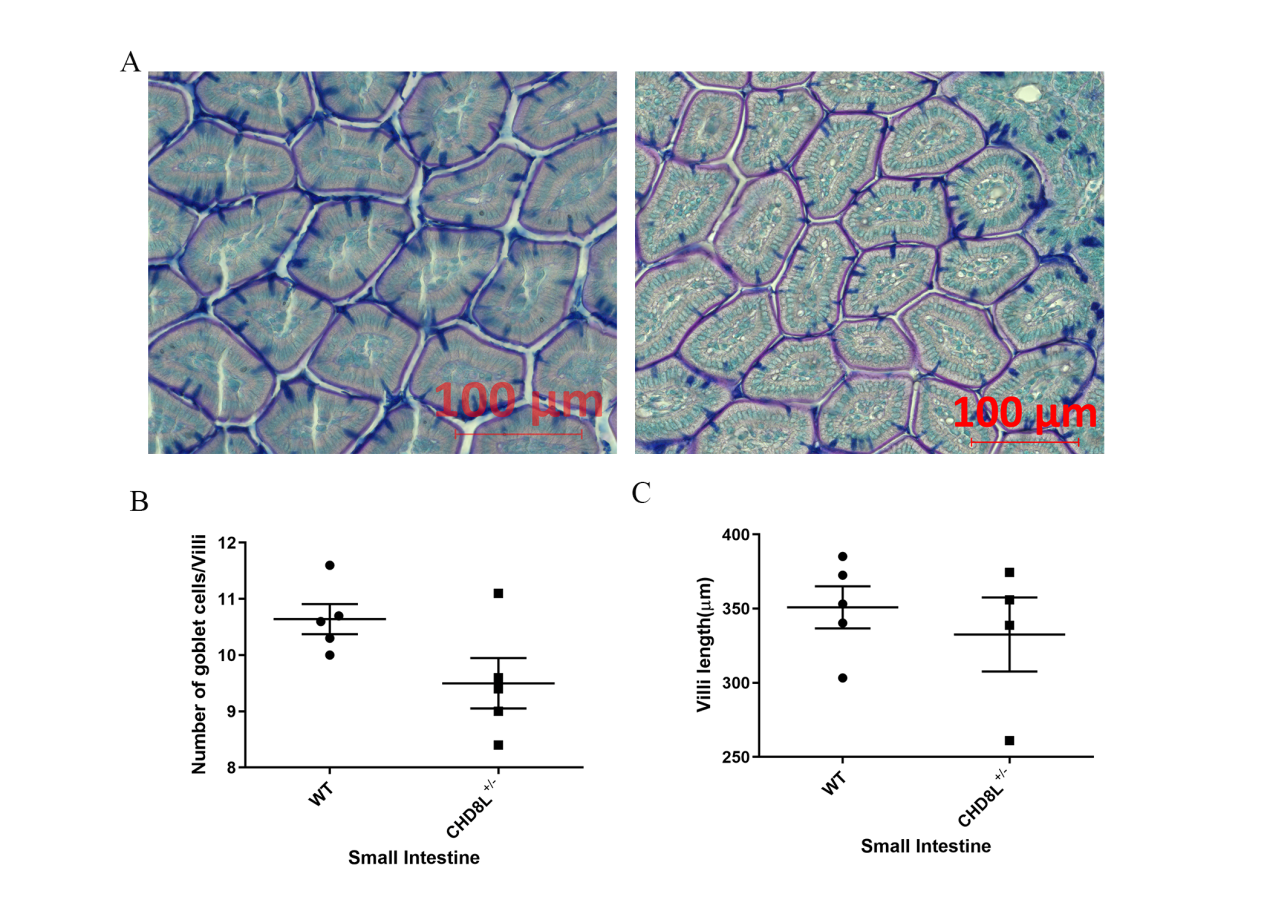
**

**A.** Representative periodic acid Schiff of gut epithelial layer from 4-week-old WT and *Chd8L*^+/-^ mice. **B.** Numbers of goblet cells per villi (p > 0.05). **C**. Villi length (p > 0.05). Data are presented as means ± SEM.

**Supplementary Figure 3.**

**
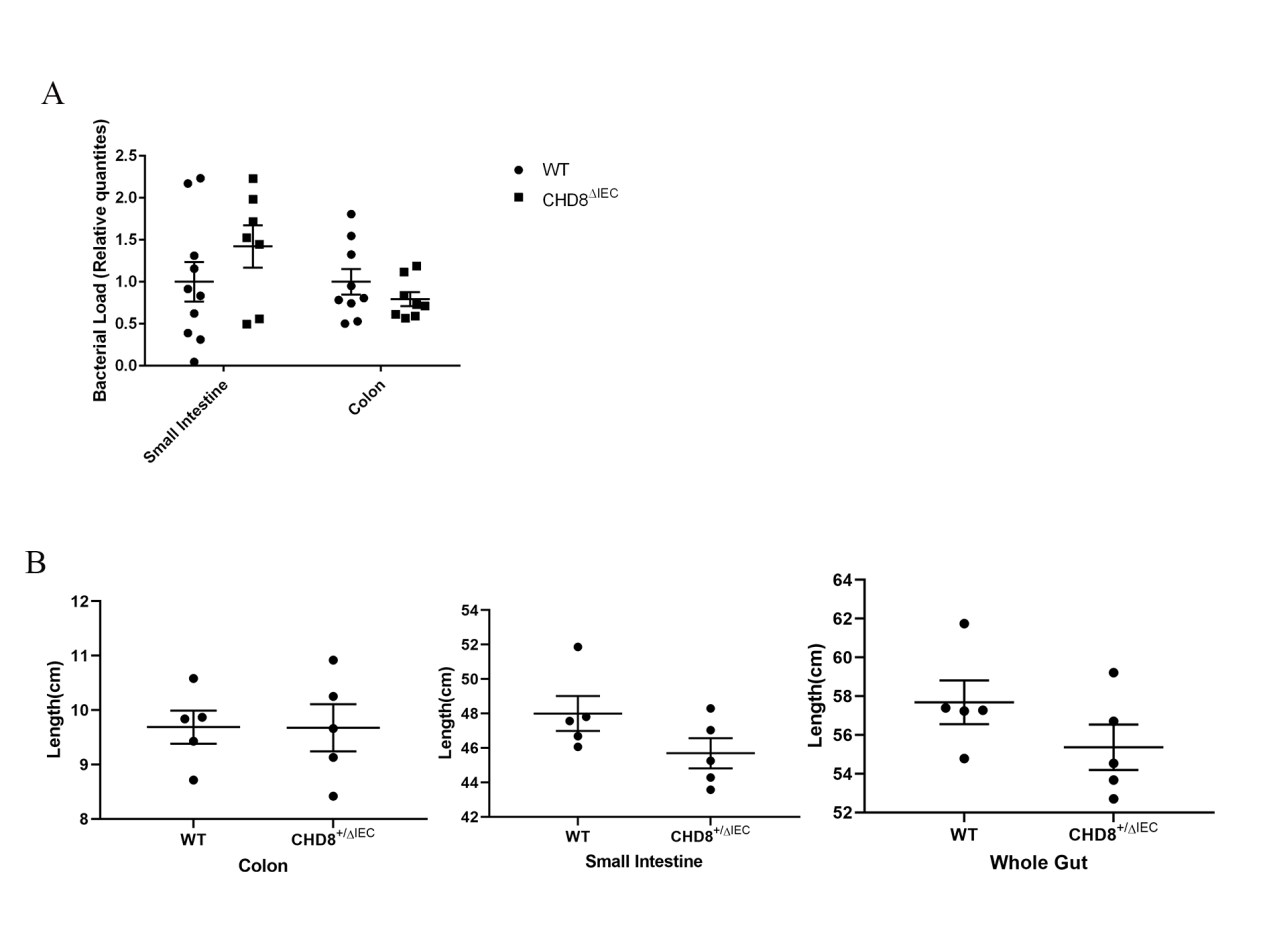
**

**A.** Relative bacterial load in the small intestine and colon of *Chd8*^+/ΔIEC^ and WT mice. (p > 0.05, two-tailed unpaired t-test; WT: n=10, *Chd8*^+/ΔIEC^, n=7). **B.** Colon, small intestine, and whole-gut lengths in WT and CHD8^+/ΔIEC^ mice (n=5 per group). Data are presented as means ± SEM.

**Supplementary Figure 4.**

**
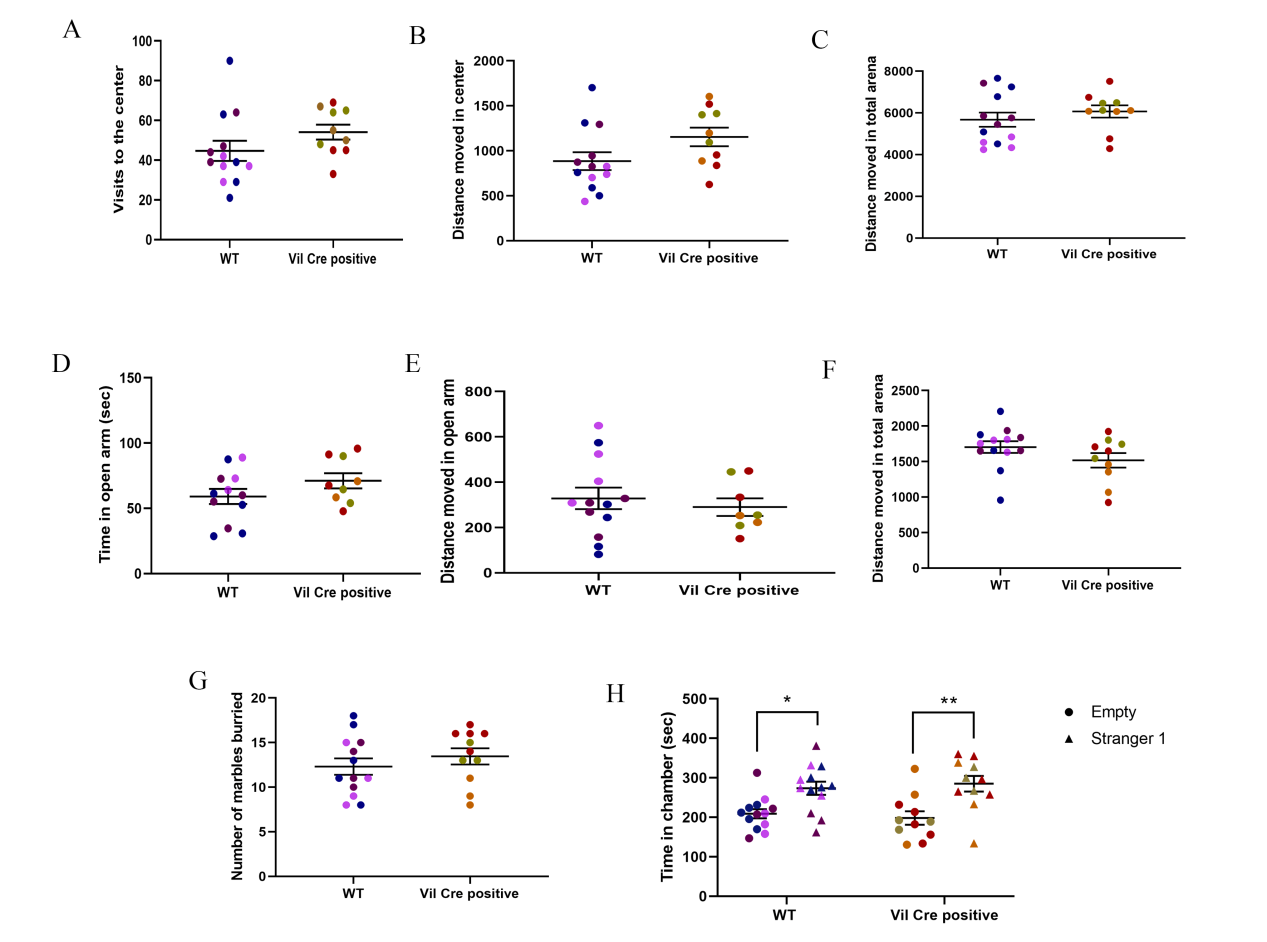
**

**A-C.** Open-field test. **A.** Visits to the center (p > 0.05; unpaired two-tailed t-test; WT: n=13, Vil cre-positive: n=10). **B.** Distance moved in the center (p > 0.05; unpaired two-tailed t-test; WT: n=13, Vil cre-positive: n=10). **C.** Distance moved in the arena (p > 0.05). **D-F.** Elevated plus maze test. **D.** Time spent in open arms (p > 0.05; unpaired two-tailed t-test; WT: n=12, Vil cre-positive: n=9). **E.** Distance moved in open arms (p > 0.05; unpaired two-tailed t-test; WT: n=13, Vil cre-positive: n=8). **F.** Distance moved in the arena (p > 0.05; unpaired two-tailed t-test; WT: n=13, Vil cre-positive: n=10). **G.** Marble burying activity (p > 0.05; unpaired two-tailed t-test; WT: n=13, Vil cre-positive: n=10). **H.** Social preference for stranger mice (*p<0.05; two-way ANOVA with Tukey’s post-hoc test; WT: n=13, Vil cre-positive: n=10). Data are presented as means ± SEM.

**Supplementary Figure 5.**

**
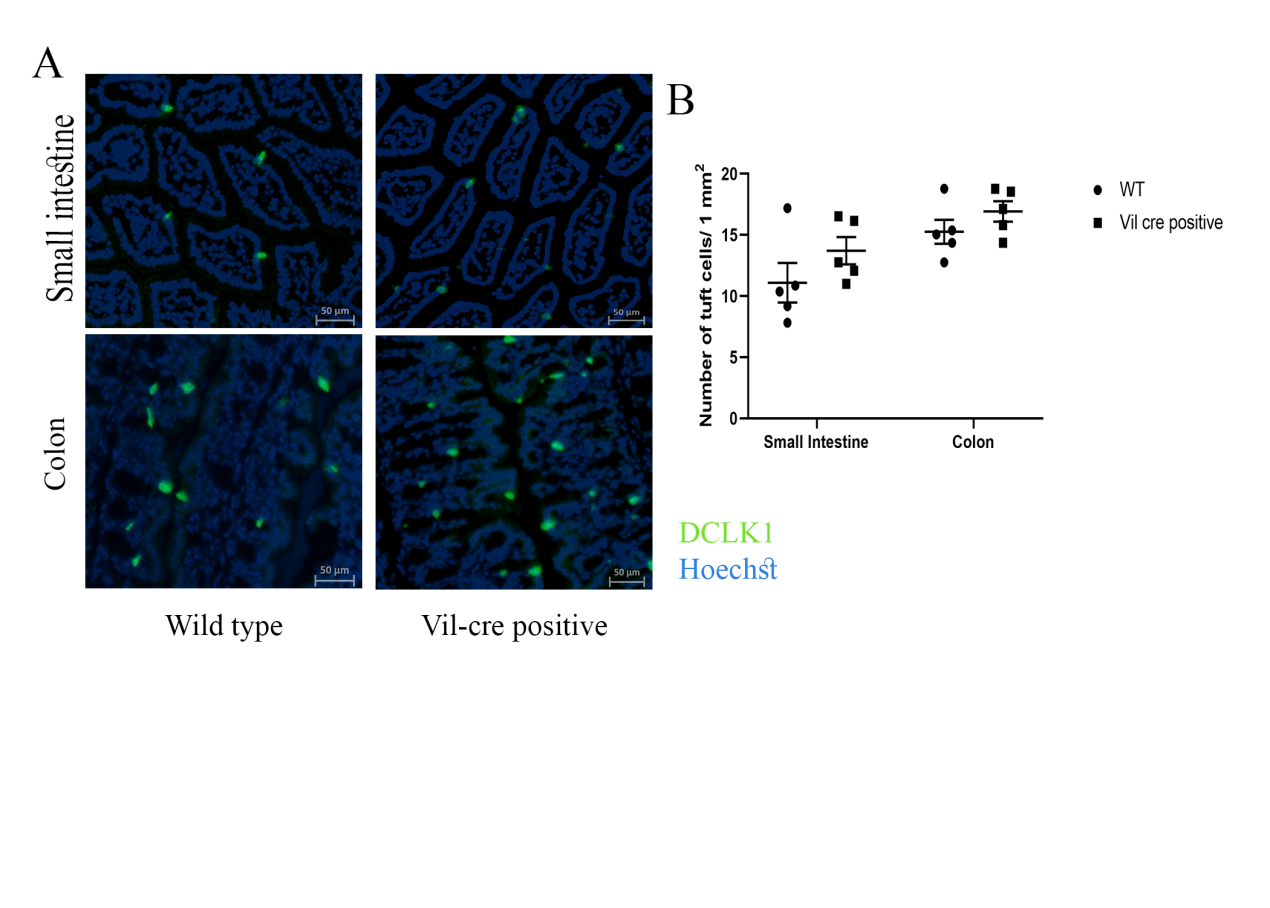
**

**A.** Representative images of DCLK1-stained small intestine and colon samples from WT and Vil cre-positive mice. **B.** Number of tuft cells per mm^2^ in the small intestines and colon. p > 0.05, n=5 for both genotypes. Data are presented as means ± SEM.
